# Role of VEGF-A and Its Receptors in Sporadic and MEN2-Associated Pheochromocytoma

**DOI:** 10.3390/ijms15045323

**Published:** 2014-03-26

**Authors:** Carla Vaz Ferreira, Débora Rodrigues Siqueira, Mírian Romitti, Lucieli Ceolin, Beatriz Assis Brasil, Luise Meurer, Clarissa Capp, Ana Luiza Maia

**Affiliations:** 1 Thyroid Section, Endocrine Division, Hospital de Clínicas de Porto Alegre, Universidade Federal do Rio Grande do Sul, Rua Ramiro Barcelos 2350, 90035-003 Porto Alegre, Rio Grande do Sul, Brazil; E-Mails: carlavazferreira@gmail.com (C.V.F.); rsiqueira.debora@gmail.com (D.R.S.); mirianromitti@gmail.com (M.R.); lu.ceolin@gmail.com (L.C.); claracapp@ig.com.br (C.C.); 2 PathologyDepartment, Hospital de Clínicas de Porto Alegre, Universidade Federal do Rio Grande do Sul, Rua Ramiro Barcelos 2350, 90035-003 Porto Alegre, Rio Grande do Sul, Brazil; E-Mails: bmassisbrasil@gmail.com (B.A.B.); meurerl@terra.com.br; (L.M.)

**Keywords:** pheocromocytoma, VEGF-A, microvessel density

## Abstract

Pheochromocytoma (PHEO), a rare catecholamine producing tumor arising from the chromaffin cells, may occurs sporadically (76%–80%) or as part of inherited syndromes (20%–24%). Angiogenesis is a fundamental step in tumor proliferation and vascular endothelial growth factor (VEGF-A) is the most well-characterized angiogenic factor. The role of angiogenic markers in PHEO is not fully understood; investigations were therefore made to evaluate the expression of VEGF-A and its receptors in PHEO and correlate to clinical parameters. Twenty-nine samples of PHEO were evaluated for VEGF-A, VEGF receptor-1 (VEGFR-1) VEGFR-2 expression and microvessel density (MVD) by immunohistochemistry. Clinical data were reviewed in medical records. The mean age of patients was 38 ± 14 years, and 69% were woman. VEGF-A, VEGFR-1 and VEGFR-2 staining were detected in nearly all PHEO samples. No significant correlation was observed between VEGF-A, VEGFR-1, VEGFR-2 expression or MVD and age at diagnosis, tumor size or sporadic and hereditary PHEO. However, the levels of expression of these molecules were significantly higher in malignant PHEO samples (*p* = 0.027, *p* = 0.003 and *p* = 0.026, respectively).VEGF-A and its receptors were shown to be up-regulated in malignant PHEO, suggesting that these molecules might be considered as therapeutic targets for unresectable or metastatic tumors.

## 1. Introduction

Pheochromocytoma (PHEO) is a rare catecholamine producing tumor arising from the chromaffin cells of the adrenal medulla (85% of cases) or extra-adrenal paraganglia [1,2]. The tumor may occur sporadically or, in approximately 20%–24% of cases, as part of the inherited syndrome. The hereditary PHEO appears as a component of multiple endocrine neoplasia type 2 (MEN 2), von Hippel–Lindau disease (VHL), neurofibromatosis type 1 (NF1) or familial paraganglioma syndromes (caused by mutations of *SDHD* and *SDHB* genes) [3–5]. The clinical picture usually results from excess secretion of catecholamines. Metastases may be present in about 14%–35% of cases [6–8].

Tumor development and its survival depends on an adequate supply of oxygen and nutrients [9]. Angiogenesis, the development of new blood vessels from established vasculature, provides growth and hematogenous dissemination of the cancer cells [10]. Several pro-angiogenic and anti-angiogenic molecules are involved in the regulation of this process [11]. Of them, vascular endothelial growth factor (VEGF; VEGF-A) is the most well-characterized angiogenic factor [9].

VEGF-A, a cytokine that exerts a critical role in both pathologic and physiologic angiogenesis, binds and activates two tyrosine kinase receptors: vascular endothelial growth factor receptor 1 (VEGFR-1; Flt-1) and vascular endothelial growth factor receptor 2 (VEGFR-2; KDR; Flk-1) [12]; on binding to its receptors, VEGF-A initiates a cascade of signaling events resulting in the activation of downstream proteins, including mitogen-activated protein kinase (MAPK) and phosphatidylinositol 3-kinase (PI3K) pathways [13,14]. Several studies have demonstrated that *VEGF-A* mRNA is upregulated in different human tumors, including lung, breast, gastrointestinal tract and kidney [15]. Furthermore, *VEGF-A* expression has been associated with poor prognosis in human tumors [16–18].

Pheochromocytomas are well-vascularized tumors but the role of VEGF-A and its receptors is poorly understood. Takekoshi *et al.* observed increased levels of VEGF-A and its receptors in 11 tumor specimens and suggested that upregulation of these molecules may be important in PHEO pathogenesis [19]. Moreover, associations between the intensity of VEGF-A expression and microvessel density (MVD) in tumor tissue and malignant phenotype have been reported [20,21]. Others studies confirmed the relationship between VEGF-A expression and malignancy, but found no association with MVD [20,22,23]. However, these studies did not evaluate the VEGF-A expression according the hereditable characteristics. In addition, other markers of angiogenesis as VEGFR-1 and VEGFR-2 were not evaluated.

The PHEO-associated MEN 2 syndrome includes two clinically distinct forms: MEN 2A and MEN 2B. Patients with MEN 2A develop medullary thyroid carcinoma (MTC), PHEO, and/or primary hyperparathyroidism (HPT). MEN 2B patients have MTC, PHEO, ganglioneuromas of the digestive tract, mucosal neuromas, and/or skeletal abnormalities [24]. The *RET* proto-oncogene, the susceptibility gene for MEN 2, encodes a receptor tyrosine kinase [25]. In MEN 2-associated MTC samples, an overexpression of the VEGF-A and its receptors was demonstrated, suggesting that these molecules might be implicated in tumor progression [26].

Surgery is the treatment of choice for the majority of patients. However the procedure might not be possible in patients with large tumors and malignant disease. Chemotherapy and radiotherapy have limited response rates and the management of advanced disease is challenging [27,28]. Currently, molecular targeted therapies are considered as the most promising strategies for metastatic disease. These therapies are designed to target a specific molecule, such as VEGF-A and RET receptors. Here, we analyzed the expression of VEGF-A, VEGFR-1 and VEGFR-2 and MVD by immunohistochemistry in samples of sporadic, malignant and MEN 2-associated PHEO.

## 2. Results

### 2.1. Patients

The clinical features and immunohistochemical staining properties of the 29 patients with PHEO are summarized in Table 1. Fourteen patients had a MEN2-associated PHEO. The mean age was 42 ± 12.7 years and 64.3% were women. Bilateral PHEO occurred synchronously or metachronously in 4 (13.8%) cases. The identified RET germline mutations in MEN2-associated PHEO patients were as follows: 7 (50%) C634Y, 1 (7.1%) C634R, 3 (21.4%) C634W, 1 (7.1%) C618R and 2 (14.3%) M918T.

Fifteen patients had sporadic disease. The mean age was 34.3 ± 14.6 years and 73.3% were women. None presented family history of PHEO or clinical features suggestive of known PHEO-associated syndromes. Four patients had metastatic disease: one patient had regional lymph node metastasis, and three had extensive invasion of adjacent structures.

### 2.2. Expression of Vascular Endothelial Growth Factor (VEGF)-A, Vascular Endothelial Growth Factor Receptor (VEGFR)-1 and VEGFR-2 in Pheochromocytomas

**Figure 1.**
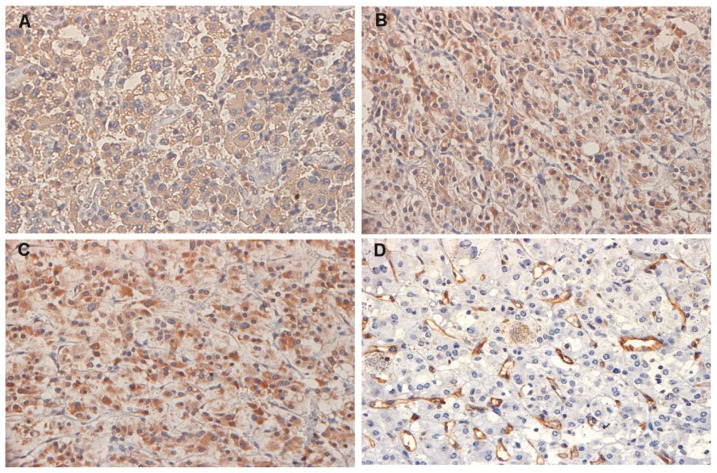
Representative immunohistochemical detection of vascular endothelial growth factor (VEGF-A), vascular endothelial growth factor receptor 1 and 2 (VEGFR-1 and VEGFR-2) and microvessels in tumor cells from PHEO samples (all images were acquired using a magnification of 400×). (**A**) VEGF-A in a malignant tumor sample; (**B**) VEGFR-1 and (**C**) VEGFR-2 in a sample of sporadic tumor; and (**D**) CD31-positively stained microvessels in a sample of MEN 2B-associated PHEO.

VEGF-A and VEGFR-1 staining were detected in all PHEO tissue samples, whereas VEGFR-2 expression was present in approximately 80% of the cases. The median of the VEGF-A immunoreactivity observed was 122 pixels (23.6–319) while to VEGFR-1 and VEGFR-2 the median were 75.9 pixels (16.6–253) and 28.8 pixels (0–338), respectively (Figure 1). These markers are also expressed in normal adrenomedullary cells (*n* = 4). The mean expression of VEGF-A and VEGFR-2 was 124.9 pixels (±37.8) and 40.3 pixels (±25.5) respectively, while the median of VEGFR-1 was 36.2 pixels (31.1–97.9). Interestingly, no significant differences were observed in the VEGF-A, VEGFR-1 or VEGFR-2 levels between normal adrenomedullary cells and benign PHEO samples (all *p* > 0.10). In normal and tumoral tissues, positive staining of VEGF-A, VEGFR-1, and VEGFR-2 were detectable in the cytoplasm of adrenal cells.

**Figure 2.**
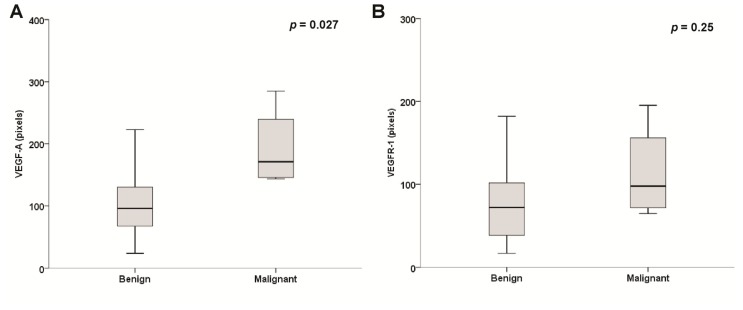

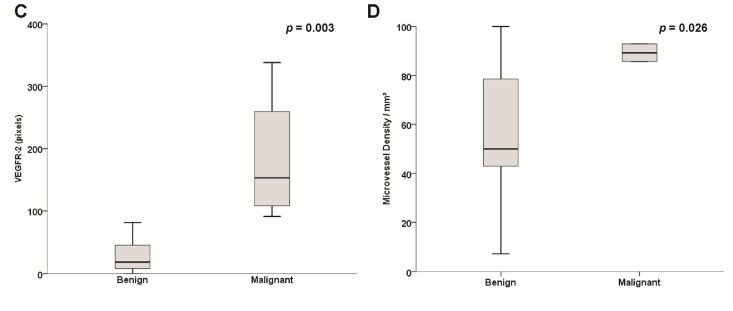
Immunohistochemical intensity of (**A**) VEGF-A; (**B**) VEGFR-1; (**C**) VEGFR-2; and (**D**) MVD in benign and malignant PHEO samples.

No statistically significant correlation was detected between VEGF-A, VEGFR-1 or VEGFR-2 expression and age at diagnosis (*r* = 0.13, *p* = 0.50; *r* = −0.09, *p* = 0.62 and *r* = −0.11, *p* = 0.55, respectively) or tumor size (*r* = 0.29, *p* = 0.14; *r* = 0.16, *p* = 0.42 and *r* = 0.13, *p* = 0.52, respectively). However, the VEGF-A and VEGFR-2 expression in malignant PHEO samples displayed significantly higher levels compared with the benign form of the disease (171 pixels (143.5–284) *vs.* 93.3 pixels (23.6–319), *p* = 0.027; 153.1 pixels (91.3–338.1) *vs.* 18.2 pixels (0–135), *p* = 0.003, respectively; Figure 2A,C). No differences were observed between the levels of VEGFR-1 between the groups (97.65 pixels (64.6–125.2) *vs.* 71.9 pixels (16.6–253), *p* = 0.25; Figure 2B).

### 2.3. Microvessel Density (MVD) Assessment

We did not observe any correlation between MVD and the expression of VEGF-A or its receptors in PHEO specimens. In addition, the analysis of the clinicopathological findings did not show a correlation between MVD and age at diagnosis or tumor size (all *p* > 0.20). However, MVD was significantly higher in malignant PHEO samples as compared with benign PHEO samples (89.3 ± 4.12 *vs.* 56.3 ± 26.2, *p* = 0.026, respectively; Figure 2D).

### 2.4. Sporadic and Hereditary Pheochromocytoma (PHEO) Tumors

**Figure 3.**
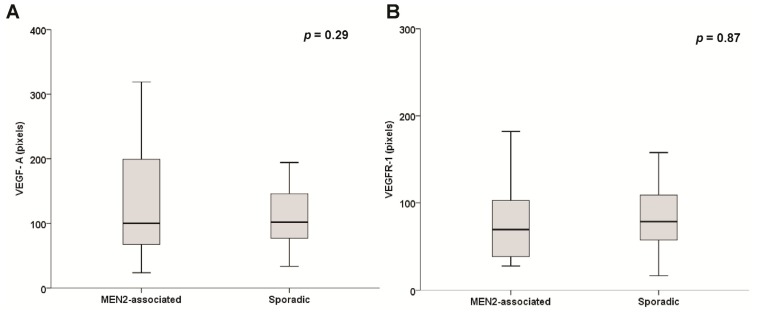

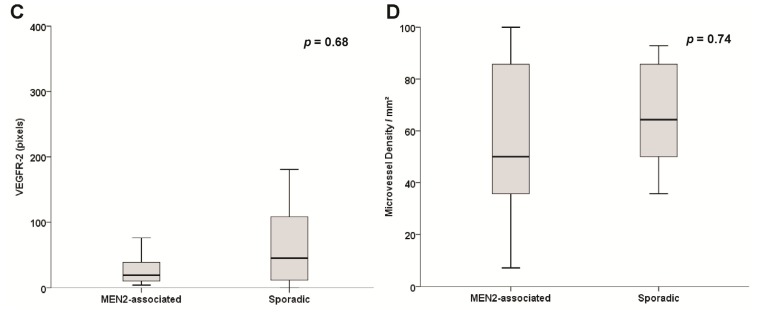
Immunohistochemical intensity of (**A**) VEGF-A; (**B**) VEGFR-1; (**C**) VEGFR-2; and (**D**) MVD in MEN2-associated and sporadic PHEO samples.

The expression of VEGF-A, VEGFR-1, VEGFR-2 and MVD were similar in sporadic and hereditary PHEO (all *p* > 0.20; Figure 3). The malignant pheochromocytoma samples were excluded from these analyses.

## 3. Discussion

In this study, we have evaluated the expression of angiogenic markers in samples of hereditary or sporadic PHEO. We observe VEGF-A and VEGFR-1 staining in all PHEO samples, whereas VEGFR-2 expression was present in the vast majority of the cases. None of these molecules were associated with the clinical presentation of PHEO. However, the expression of VEGF-A and VEGFR-2 was up regulated in malignant PHEO samples.

Patients with PHEO often experience debilitating symptoms and may have a poor quality of life, due to excess catecholamine secretion. The main symptoms and signs of PHEO include hypertension, palpitations, headache, sweating and pallor. According to the degree of catecholamine excess, patients may present with myocardial infarction, arrhythmia, stroke or other vascular manifestations [27,28]. Surgery is the treatment of choice for PHEO. However, in metastatic disease curative resection is seldom possible. In these cases, chemotherapy and radiotherapy have been proposed; but, unfortunately, both have limited value [27,29]. The diagnosis of malignancy in PHEOs is often difficult because of the lack of reliable histological or molecular markers; according to the World Health Organization, the only criterion to diagnose malignant PHEO is the presence of metastasis at non-chromaffin sites. For patients with metastatic disease, the five-year overall survival rate ranges from 40% to 77% [30,31]. Thus, the identification of clinical, histopathological or immunohistochemical parameters able to determine a potentially malignant tumor early, before the onset of clinically evident metastases, could increase the chances of cure.

Previous studies showed increased expression of VEGF-A and its receptors in PHEO and demonstrated a correlation between VEGF-A expression and malignant phenotype [19–21]. Here, we also observed increased levels of VEGF-A and VEGFR-2 in malignant PHEO, further supporting the idea that these molecules may help in the identification of PHEO with malignant behavior. The better understanding of different signaling pathways and multiple genetic abnormalities involved in the pathogenesis of cancer has allowed the development of targeted molecular therapies. Among the novel agents are the protein tyrosine kinase inhibitors. These are small molecules that inhibit different tyrosine kinase receptors, including VEGFR-1, VEGFR-2. Indeed, it has been reported that sunitinib treatment induced complete or partial responses in patients with malignant PHEOs and paragangliomas [32,33]. Interestingly, it was suggested that sunitinib treatment can be involved in direct cytotoxic effects in PHEO cells [34]. The results presented here further support the use of tyrosine kinase inhibitors as a rational therapeutic target in tumors that develop from chromaffin cells.

Considering the differences between the pathogenesis of hereditary and sporadic PHEO, mainly the presence of germline mutation in inherited forms, it is reasonable to investigate if these issues could interfere with tumoral angiogenesis. In patients with MTC-related MEN2, the analysis of angiogenic factors found that patients with hereditary form display increased expression of VEGFR-1, whereas higher expression of MVD was observed in sporadic MTC [26].The VEGF-A and its receptors mRNA levels were evaluated in a large cohort of PHEO (102 patients with sporadic PHEO and 86 with hereditary form); the samples were separated into two groups depending on the presence of activation of the pseudo-hypoxic pathway. It was observed that PHEOs associated with *SDHx* mutations and VHL disease had a markedly increase in the expression of major angiogenic molecules than those associated with NF1 disease, MEN2 syndrome or sporadic tumors. The expression level of VEGF-A in hereditary paraganglioma associated with inactivation of the *SDHD* gene was higher than those observed in sporadic tumors [35]. Here, we show that there are no differences in VEGF-A and its receptors expression between sporadic and MEN2-associated PHEO. These findings suggest that tumor angiogenesis may vary according to the molecular cause of the tumor [36].

In the same way as the VEGF-A expression, tumoral vascular density has been studied as prognostic factor. Several studies have associated a high number of vessels in human tumors with the presence of metastasis [37–39]. We did not observe an association between MVD and clinical or pathological features of PHEO, but we showed an association between malignant PHEO and vascular density. Previous studies on MVD in PHEO described discrepant results. First, Liu *et al.* correlated the number of tumor blood vessels with the invasive behavior [40], however this correlation was not later, confirmed by Ohji *et al.* [23]. Our results are in accordance with recent reports that demonstrated higher MVD in malignant PHEO samples [21,22,41]. Possible explanations for these discrepancies include differences in techniques for MVD counting as well as differences among the populations studied.

PHEOs are rare tumors of the adrenal medulla; for this reason it is difficult to perform studies with large samples. Therefore, an important limitation of our study is the small sample size, which may explain the absence of significant findings between VEGF-A and its receptors and clinical and oncological features (type 2 error). In addition, we excluded the presence of other hereditary forms of PHEO only through clinical data, physical examination and family history; unfortunately, we did not have material available for genotyping all the germline mutations in the susceptibility genes of the PHEO.

## 4. Material and Methods

### 4.1. Patients

We have studied tumor specimens from 29 patients with a diagnosis of PHEO attended in the Endocrine Division at Hospital de Clínicas de Porto Alegre (Porto Alegre, Rio Grande do Sul, Brazil), a tertiary care, university-based teaching hospital. Our Institution is a referral center for molecular diagnosis of hereditary MTC. Fourteen patients had MEN 2-associated PHEO, whereas fifteen had apparently sporadic PHEO (sPHEO). All patients with MEN2-associated PHEO harbor a *RET* germline mutation, identified by standard procedures described previously [42]. For those with sPHEO, the diagnosis was established based on the absence of known *RET* germline mutation, family history of PHEO and/or clinical phenotype of a specific syndrome. According to the World Health Organization (WHO), malignant PHEO is defined by the presence of metastases at non-chromaffin sites distant from the primary neoplasm (such as bone, liver or lungs); however, a significant invasion is considered by some pathologists as the sign of malignancy [28]. Here we considered malignant PHEO for patients with metastatic disease or extensive invasion of surrounding structures.

The data collected for each individual included the clinical and histopathological characteristics of PHEO, the association of another endocrine neoplasia, the presence of affected family members and the presence of *RET* germline mutations.

We evaluated also four normal adrenal glands obtained from patients submitted for nephrectomy due to renal diseases.

The study protocol was approved by the Institutional Ethics Committee.

### 4.2. Immunohistochemistry Analysis (IHC)

Immunohistochemistry analysis (IHC) was performed on thin sections (3 μm) of previously formalin-fixed and paraffin-embedded tissues. The antibodies used were polyclonal rabbit anti-human VEGF-A (clone VG1; ab1316; Abcam Inc., Cambridge, MA, USA), monoclonal mouse anti-human VEGFR-1 (361100; Invitrogen, Frederick, MD, USA), and monoclonal mouse anti-human VEGFR-2 (A-3: SC-6251; Santa Cruz Biotechnology, Santa Cruz, CA, USA). Sections representing PHEO were submitted to routine immunohistochemical technique, which comprises deparaffination and rehydration, antigenic recovery, inactivation of endogenous peroxidase, and blockage of unspecific reactions. Primary antibodies were incubated overnight at a temperature of 4 °C, at dilutions of 1:100 (VEGF-A), 1:100 (VEGFR-1), and 1:200 (VEGFR-2), followed by application of streptavidin horseradish peroxidase conjugate (LSAB; DakoCytomation, Via Real Carpinteria, CA, USA), and diaminobenzidinetetrahydrochloride (Kit DAB; DakoCytomation). Sections of human tissue were used as a positive control (skeletal muscle tissue for VEGF-A, placenta for VEGFR-1 and intestinal tumor for VEGFR-2), and absence of the primary antibody as a negative control.

### 4.3. Semi-Quantitative Analysis for the Intensity of Positive Staining in Tissues

The intensity of positive staining of VEGF-A, VEGFR-1, and VEGFR-2 was performed by digital image analysis using the Image-Pro Plus 6.0 software (Media Cybernetics, Rockville, MD, USA). Two independent researchers analyzed the intensity of brownish-colored immunostaining in pixels in 10 fields from each slide. We used the mean number of pixels identified by both researchers in each sample. All of the images were taken using the same microscope and camera sets [43].

### 4.4. Microvessel Density (MVD) Assessment

For evaluation of MVD as a measure of angiogenesis, samples were prepared for IHC, as described above, using primary anti-CD31 antibody (clone JC7OA, M0823; DakoCytomation), at dilutions of 1:200. Sections of human lung carcinoma were used as positive control and omission of the primary antibody as a negative control.

The Chalkley point technique was used for assessment of vascular density internationally acknowledged as the criterion standard for the evaluation of MVD [44]. The densest vascular areas (known as hot spots) were determined at low magnification (×100). For each tissue sample, two or three hotspots were selected, depending on the size of the tumor. The mean of the counts for the most angiogenic areas (hot spot) was recorded at ×400 magnification. The Chalkley point count was performed by two observers independently. The final MVD was the mean value of the two independent counts.

The results of MVD are expressed per one mm^2^ (×400; 0.14 mm^2^ per field).

### 4.5. Statistical Analysis

Results are expressed as mean and standard deviation or median and minimum and maximum, accordingly the variable distribution. Spearman’s coefficient test was used to assess the correlation between expression of the angiogenic markers (VEGF-A, VEGFR-1, or VEGFR-2) and MVD with age at surgery and tumor size. Mann-Whitney U-test was used to compare angiogenic markers and MVD between patients with sporadic or hereditary and malignant disease. The Statistical Package for the Social Sciences 18.0 (SPSS Inc., Chicago, IL, USA) was used and *p* < 0.05 was considered as statistically significant.

## 5. Conclusions

We observed high levels of expression of VEGF-A and its receptors in malignant PHEOs. These molecules are involved in the tumorigenesis process and might be considered as potential therapeutic targets for new drugs, such as tyrosine kinase inhibitors, in patients with metastatic PHEO. These findings also suggest that VEGF-A, VEGFR-2 and MVD are potential markers for the diagnosis of malignant PHEO samples even before the onset of clinically evident metastases.

## Figures and Tables

**Table 1.** Clinical features and vascular endothelial growth factor (VEGF)-A, VEGF receptors expression and microvessel density (MVD) in pheochromocytoma (PHEO) patients (*n* = 29).

Case	Phenotype	RET germline mutation	Age (years) 1/sex	Tumor size 2	VEGF-A 3	VEGFR-1 3	VEGFR-2 3	MVD 4
1	MEN 2 A	C618R	38/F	7.50	118.6	102.8	16	78.6
2	MEN 2 A	C634W	36/F	4.0	23.6	54	6.5	100
3	MEN 2 A	C634R	37/F	6.40	319	27.6	4.2	35.7
4	MEN 2 A	C634R	21/F	1.70	63.2	38.3	19.2	14.3
5	MEN 2 A	C634R	34/F	5.50	78.5	182	29.6	92.9
6	MEN 2 B	M918T	49/F	N/A	95.9	75.9	N/A	85.7
7	MEN 2 B	M918T	33/F	2.5	67.3	47.6	67.6	50
8	MEN 2 A	C634Y	61/M	N/A	199.1	35.5	38.7	50
9	MEN 2 A	C634Y	44/F	1.00	97.2	81.1	17.3	7.14
10	MEN 2 A	C634Y	49/M	1.20	222.9	101.6	75.9	7.14
11	MEN 2 A	C634Y	55/M	N/A	102.6	163	52.2	57.1
12	MEN 2 A	C634Y	62/F	3.50	220.1	35.8	4.5	42.9
13	MEN 2 A	C634Y	45/M	2.5	65.8	62.7	10.3	92.8
14	MEN 2 A	C634Y	23/M	1.4	112.5	253.1	33.2	50
15	Sporadic 5		14/F	6.50	143.5	64.6	338.1	92.8
16	Sporadic 5		30/F	8.20	284.8	116.7	180.8	92.9
17	Sporadic 5		23/M	13.0	194.1	195.2	91.3	85.7
18	Sporadic 5		52/M	4.0	147.9	78.6	125.5	85.7
19	Sporadic		20/F	5.50	130.1	37.3	45.1	64.3
20	Sporadic		18/M	6.00	76	50	1.6	50
21	Sporadic		34/F	11.5	89.4	87.9	16	50
22	Sporadic		45/F	4.00	135.7	229.7	81.4	42.9
23	Sporadic		39/F	5.50	51.4	68.5	13.4	85.7
24	Sporadic		23/F	3.50	33.4	71.9	0	92.8
25	Sporadic		23/F	8.50	77.4	92.5	135	42.9
26	Sporadic		35/F	11.0	160.6	157.7	28.1	35.7
27	Sporadic		38/F	7.50	101.8	16.6	2.3	57.1
28	Sporadic		61/F	3.5	44	101.2	45.3	64.3
29	Sporadic		58/M	3.4	81.4	28.5	9.9	57.1

N/A, no available;

1 age at diagnosis of PHEO;

2 Greatest tumor diameter (cm);

3 Pixels;

4 MVD/mm^2^; and

5 MalignantPHEO.
